# CD19 and CD70 Dual-Target Chimeric Antigen Receptor T-Cell Therapy for the Treatment of Relapsed and Refractory Primary Central Nervous System Diffuse Large B-Cell Lymphoma

**DOI:** 10.3389/fonc.2019.01350

**Published:** 2019-12-04

**Authors:** Sanfang Tu, Xuan Zhou, Zhenling Guo, Rui Huang, Chunyan Yue, Yanjie He, Meifang Li, Yiran Chen, YuChen Liu, Lung-ji Chang, Yuhua Li

**Affiliations:** ^1^Department of Hematology, Zhujiang Hospital, Southern Medical University, Guangzhou, China; ^2^Department of Research and Development, Geno-Immune Medical Institute, Shenzhen, China; ^3^School of Medicine, University of Electronic Science and Technology of China, Chengdu, China; ^4^Department of Molecular Genetics and Microbiology, College of Medicine, University of Florida, Gainesville, FL, United States

**Keywords:** chimeric antigen receptor (CAR), central nervous system (CNS), diffuse large B-cell lymphoma (DLBCL), CD19, CD70

## Abstract

**Background:** The therapeutic efficacy of chimeric antigen receptor (CAR) T-cells targeting CD19 has been illustrated in the treatment of diffuse large B-cell lymphoma (DLBCL). However, there is a 21–35% relapse rate after anti-CD19 CAR T-cell induced remission. In addition, CAR T-cell therapy has severe adverse reactions, such as cytokine release syndrome (CRS) and CART-related encephalopathy syndrome (CRES). Because of the potential mortality associated with severe CRES, patients with primary central nervous system lymphoma (PCNSL) are usually excluded from clinical trials involving CAR T-cell therapy. Here, we report a case of refractory and relapsed primary central nervous system diffuse large B-cell lymphoma (PCNS-DLBCL).

**Case Presentation:** The patient is a 67-year-old male who was diagnosed with PCNSL in 2011. He achieved complete remission (CR) after receiving 6 cycles of temozolomide and high-dose methotrexate. In December 2016, he experienced his first relapse and was treated with surgery and multicourse chemotherapy. He achieved CR again after the treatment. However, he experienced a second relapse in August 2017. MRI revealed a residual mass of 26 mm^*^35 mm^*^30 mm on the right side of the post-operative cavity and stale hemorrhage in the left basal ganglia. After confirming the expression of CD19 and CD70 in his tumor samples, the patient was given lymphodepletion chemotherapy followed by infusion of 4th generation CD19-CAR T-cells (4SCART19) and 4th generation CD70-CAR T-cells (4SCART70). One month later, the patient had symptomatic improvement, and brain MRI showed CR. Both CART19 and CART70 cells were detected in the 10th month after CAR T-cell infusion. Notably, neither CRS nor CRES occurred during treatment and follow-up. To date, the patient has maintained disease-free survival with more than 17 months of follow-up.

**Conclusions:** The results of this study indicate that combination of CD19- and CD70-specific CAR T-cells may effectively target PCNSL and maintain disease-free survival without inducing CRS or CRES. Therefore, central nervous system lymphoma is not an absolute contraindication for dual-target CAR T-cell therapy.

## Background

Primary central nervous system lymphoma (PCNSL) is a rare but highly malignant extranodal type of non-Hodgkin lymphoma, which accounts for ~3% of CNS tumors (1, 2). About one-third of patients with PCNSL appear to be resistant to first-line treatment, and half of the patients who have achieved remission will relapse (3). CAR T-cells targeting CD19 is a revolutionary immunotherapy in treating relapsed or refractory (R/R) B lineage malignancies (4) and has been reported to induce a 64–86% response rate in patients with DLBCL (5). However, CAR T-cell therapy remains controversial due to safety concerns. The incidence of cytokine release syndrome (CRS) or CART-related encephalopathy syndrome (CRES) in patients with hematological malignancies is significantly higher than that in patients with other solid malignancies (6). The incidence of CRES is reported to range from 19 to 64%, and the incidence of severe CRES (grade ≥3) is reported to range from 12 to 28% (7–10). Due to the potential mortality associated with CRES, patients with PCNSL are excluded from almost all clinical trials of CAR T-cell therapy. There are few reports on the application of CD19-targeted CAR T-cells for central nervous system lymphoma (11). In addition, the high relapse rate, especially that of CD19-negative relapse, remains another problem to be solved (12). A potential strategy to prevent relapse due to antigen escape is to infuse T-cells capable of recognizing multiple antigens (13). CD70 is a promising therapeutic target due to its restricted expression in normal tissues and its overexpression in lymphoma tissues (14). In addition, it has been reported that anti-CD70 CAR T-cell therapy eliminated primary CD70-positive cells and had strong anti-tumor effects in preclinical animal models (15, 16).

The CAR T-cells used in our center are fourth-generation CAR (4SCAR) T-cells, and the double CART treatment involved infusion of CART19 and CART70 cells, respectively. As Figure S1 show, the 4SCART70 is composed of Secretory signal peptide, CD70 antigen binding domain, CD28 transmembrane domain, CD28 extracellular signal transduction domain, CD28 intracellular signal transduction domain, CD27 intracellular signal transduction domain, CD3 Zeta intracellular signal transduction domain, 2A sequence and inducible switch (iCasp9), which are arranged as follows: Secretory-CD70 scFv-CD28-CD27-CD3z-2A-iCasp9. The structure of 4SCART19 is similar to that described above. Compared to the second- or third-generation CAR T-cells, the embedding of the suicide gene (iCasp9) contributes to the elevated safety of 4SCAR T-cells. 4SCAR T-cells can be depleted when uncontrollable toxicity is observed following CAR T-cell infusion. The details of the CART manufacturing process are as previously described (17). The safety and efficacy of 4SCAR T-cells have been demonstrated in patients with highly resistant B-cell lymphoma (18, 19). No CRS response greater than grade 2 was observed in 35 of the 36 patients treated with single or multitarget 4SCAR T-cells. In addition, no neurotoxicity was observed (19).

To explore the safety and effectiveness of dual-target CAR T-cells in the treatment of PCNS-DLBCL, we conducted a clinical trial utilizing 4SCAR T-cells specifically targeting against both CD19 (4SCAR19) and CD70 (4SCAR70). Here, we report a case of R/R PCNSL that achieved long-term disease-free survival due to the therapy using a combination of CD19- and CD70-specific fourth-generation CAR T-cells.

## Case Report

**Figure 1:**
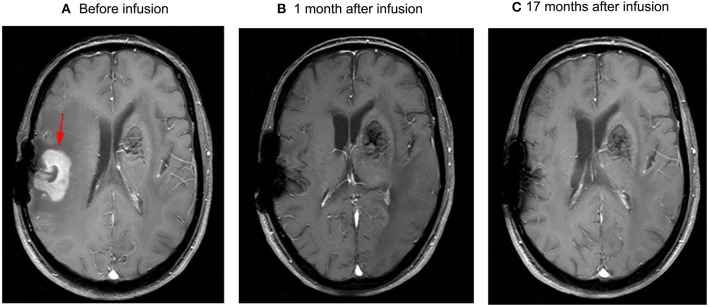
Brain MRI **(A)** the residual mass on the right side of the post-operative cavity, which was ~26 mm*35 mm*30 mm (arrow) in volume, and the mass disappeared 1 month after infusion **(B)**. No relapse occurred in subsequent follow-up **(C)**.

A 67-year-old male was diagnosed with PCNS-DLBCL in 2011. He achieved complete remission (CR) after receiving treatment with six cycles of temozolomide plus high-dose methotrexate. In December 2016, he experienced his first relapse and was treated with one course of glucocorticoids and temozolomide as well as right frontal lobe space-occupying resection. This treatment was followed by one course of rituximab and temozolomide combined with high-dose methotrexate and six cycles of rituximab and ibrutinib combined with high-dose methotrexate. He achieved CR again after the treatment. In August 2017, he had a second relapse with clinical symptoms, including dizziness, dysphagia, and distortion of commissure to the right and weakness in his left extremities. MRI suggested a residual mass (26 mm^*^35 mm^*^30 mm) on the right side of the post-operative cavity and stale hemorrhage in the left basal ganglia (Figure 1A). There were no lymphoma cells found in the cerebrospinal fluid (CSF). Immunohistochemical staining of tumor sections showed DLBCL with expression of CD19 and CD70. The patient was enrolled into a clinical trial (clinicaltrial.gov registry NCT03125577). Consent for publication in print and in electronic file was obtained from the patient. In October 2017, peripheral blood mononuclear cells were collected from the patient. The blood sample was sorted by anti-CD3 beads and then activated with anti-CD3/CD28 monoclonal antibodies before lentiviral 4SCAR infection. T-cells were transduced with a safety-engineered lentiviral vector coding a fourth-generation CAR containing anti-CD19 or anti-CD70 scFv fused with multiple intracellular signaling domains (CD28-CD27-CD3z-2A-iCasp9). The patient received lymphodepleting chemotherapy consisting of fludarabine (30 mg/m^2^/d) and cyclophosphamide (300 mg/m^2^/d) on days −4 to −2 and then infusion of 1^*^10^8^ CAR19 T-cells and 8.2^*^10^7^ CAR70 T-cells on day 0.

**Figure 2:**
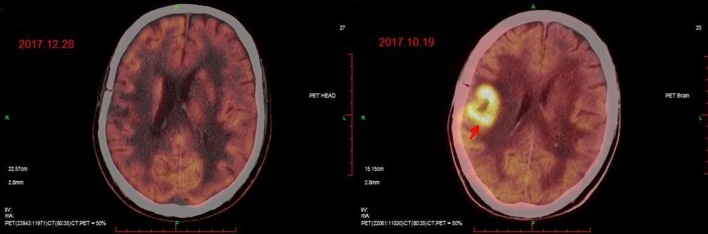
PET/CT images before **(Right)** and after **(Left)** CAR T-cell infusion. The position indicated by the arrow is the location of the lesion.

**Figure 3:**
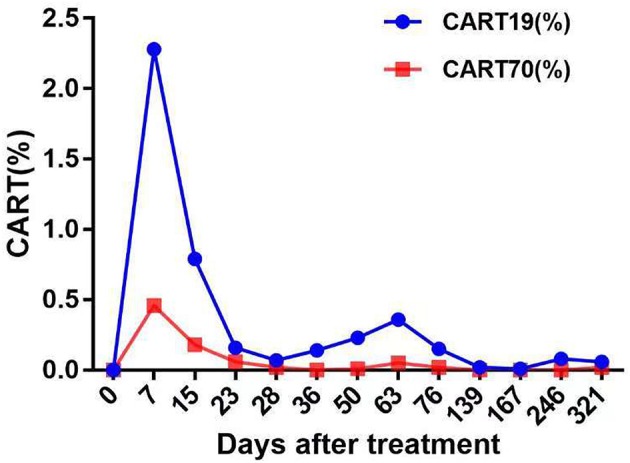
The levels of fourth-generation CART19 and CART70 cells in peripheral blood at the indicated time points after CAR T-cell infusion. Both 4SCAR19 and 4SCAR70 reached their peaks in the peripheral blood on day 7, with the former accounting for 2.28% of circulating mononuclear cells and the latter accounting for 0.46%.

**Figure 4:**
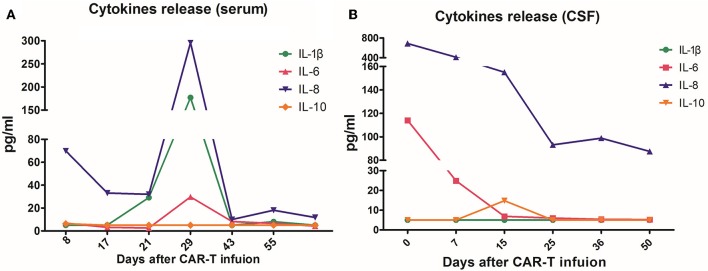
The concentrations of interleukin (IL)-1β, IL-6, IL-8, and IL-10 in plasma **(A)** and cerebrospinal fluid (CSF) **(B)** before and after CAR T-cell infusion. On day 29 after the infusion, the cytokines of the peripheral blood reached their highest peak, with IL-8 296 pg/mL and IL-1β 177 pg/mL. In CSF, both IL- 6 and IL-8 showed a downward trend after infusion, while IL-1β and IL-10 remained at low levels.

The patient had a poor appetite and felt mild fatigue for a few days after both infusions. These clinical symptoms gradually resolved in 10 days. One month after CAR T-cell infusion, restaging cranial MRI indicated that the lesion area in the right temporal parietal lobe had disappeared (Figure 1B). PET/CT imaging confirmed no space occupying mass (Figure 2). This result suggested the achievement of CR. In April 2019, 17 months later, MRI suggested that the patient had achieved durable remission (Figure 1C). Only slight hematologic toxicity was found in this case. There was no indication of CRES or CRS. The copy number of CAR in the patient's peripheral blood was determined by qPCR. The number of CAR T-cells in the peripheral blood peaked on day 7, with the 4SCAR19 in the peripheral blood accounting for ~2.28% of circulating mononuclear cells, while the 4SCAR70 was 0.46% of circulating mononuclear cells. 4SCAR19 and 4SCAR70 showed similar expansion kinetics *in vivo*. The amplification of CAR19 T-cells was more pronounced than that of the CAR70 T-cells (Figure 3). Both CAR19 and CAR70 T-cells were detectable more than 10 months after CAR T-cell infusion. The concentrations of cytokines, including IL-1β, IL-6, IL-8, and IL-10, in his plasma and CSF was tested by flow cytometry at regular intervals (Figure 4). Although IL-1b and IL-8 were evidently increased and reached their peaks on the day 29, they did not reach the levels causing severe CRS. Notably, the levels of IL-6 and IL-8 decreased in the CSF, while the levels of IL-1β and IL-10 remained stable.

## Discussion

PCNSL is a rare but highly malignant extranodal type of non-Hodgkin lymphoma, with more than 50% of patients aged 60–80 years (20). More than 90% of PCNSL patients are diagnosed as DLBCL (2), and almost half of patients are R/R. In the past few decades, the treatment of patients with PCNSL has significantly improved. It is reported that the median overall survival (OS) increased from 8 months in the 1970s to 35 months in the 2010s in elderly patients aged 50–69 years (20). However, the prognosis of primary R/R PCNSL remains poor, with a median survival of 2 months without additional treatment (21). Salvage treatment includes chemotherapy, radiotherapy, hematopoietic stem cell transplantation and novel targeted drugs (22–25). High-dose chemotherapy and autologous hematopoietic stem cell transplantation is used in patients with R/R PCNSL with a 3-year event-free survival of 53% and an OS of 64% (24). Although the tumor is sensitive to radiotherapy, whole brain radiotherapy has an unacceptable response with a median overall survival (OS) of 11–16 months (25). At present, no consensus has been reached on the treatment of R/R PCNSL, and the OS of patients with R/R PCNSL is still unsatisfactory (2, 22, 23). New alternative treatments are imperative.

CAR T-cell therapy is an attractive method for generating an anti-tumor immune response. As early as 2010, a patient experienced a dramatic regression of his lymphoma after infusion of CART19 cells (26). Cumulative data have shown that immunotherapy with CAR T-cells provides hope for a high response rate in patients with R/R DLBCL (7–10). However, the occurrence of lethal cerebral edema after CAR T-cell therapy prevents most patients with central tumor infiltration from receiving CAR T-cell therapy. Moreover, elderly patients have a high risk of developing severe neurotoxic effects associated with immunotherapy treatment. In 2017, Abramson JS reported one case of successful CART19 therapy, which induced CR in a 68-year-old woman with CNS lymphoma (11). This study suggested that a CNS tumor is not an absolute contraindication to CAR T-cell therapy. Although new strategies for CART19 treatment have been developed in recent years, 21–35% of patients still experience relapse after anti-CD19 CAR T-cell induced remission (4). Simultaneously targeting another tumor antigen to prevent relapse has been proposed (27). As the cellular ligand of the tumor necrosis factor receptor family, CD70 is expressed on a wide variety of malignancies, including 71% of large B-cell lymphomas (14). A high level of CD70 expression is related to an unfavorable outcome for DLBCL, suggesting that this molecule may constitute a potential therapeutic target in selected DLBCLs (28, 29). Moreover, it has been shown that CD70-CARTs could effectively reduce tumor burden without impairing the immune response in experimental animal models (15).

In this study, we report the case of an elderly patient with R/R PCNS-DLBCL, who suffered a second relapse and failed salvage treatment with high-dose chemotherapy. This patient was given CAR T-cell therapy targeting CD19 and CD70. The patient achieved 17 months disease-free survival without CRS, CRES or any other severe side effects. This case indicates that CAR T-cell therapy is a potentially promising treatment for patients with R/R PCNS-DLBCL. This is the first report presenting the feasibility of fourth-generation dual-target-specific CAR T-cell therapy in the treatment of PCNS-DLBCL. In this case, both 4SCAR19 and 4SCAR70 T-cells were still detectable on day 321 after CAR T-cell infusion. This result is consistent with a previous report that CAR T-cells can persist at high levels for a long time (30). The clinical symptoms of the patient were relieved by day 10, shortly after the peak expansion of both 4SCAR19 and 4SCAR70 T-cells that occurred on day 7 after infusion. A one-arm, multicenter, phase 1–2 trial has reported that 11 of the 32 evaluable patients (34%) maintained an ongoing response during follow-up for 24 months, but they no longer had detectable genetically marked CAR T-cells (31), which suggested that CR could be sustained after the disappearance of CAR T-cells in peripheral blood. The patient in our center achieved CR after 1 month and has maintained disease-free survival for 17 months after CAR T-cell therapy. It seems that the patient has a high probability of long-term disease-free survival according to the study, which suggested that achievement of complete or partial responses for 3 months might indicate long-term response durability (31).

It was reported that normal tissues of the CNS lack CD19 expression (32). CD70 expression is limited in CNS (33). To date, no serious adverse effects, such as CRS, have been reported in the preclinical studies of anti-CD70 CAR T-cell therapy (15, 34). The durable remission and absence of severe clinical toxicity in this patient indicates that the application of CAR19 T-cell therapy in combination with CAR70 T-cell therapy in patients with PCNS-DLBCL is potentially beneficial and safe.

The continued follow-up of this patient and the enrollment of more cases are needed to determine whether fourth-generation dual-target-specific CAR T-cell therapy is a therapeutic option for PCNSL.

## Data Availability Statement

The raw data supporting the conclusions of this article will be made available by the authors, without undue reservation, to any qualified researcher.

## Ethics Statement

The studies involving human participants were reviewed and approved by Zhujiang Hospital, Southern Medical University Medical Ethics Committee. The patients/participants provided their written informed consent to participate in this study.

## Author Contributions

ST and XZ wrote the manuscript. ZG contributed to the data collection. RH revised the manuscript. CY contributed to patient management. YH took part in the registration of clinical research. ML completed the production of figures. YC was responsible for the detection of CAR T-cells and cytokines. YLiu and LC performed the production of CAR T-cells. YLi directed the clinical trial. All authors read and approved the final manuscript.

### Conflict of Interest

The authors declare that the research was conducted in the absence of any commercial or financial relationships that could be construed as a potential conflict of interest.
