# Supplementary figures and images for: CD19 and CD70 Dual-Target Chimeric Antigen Receptor T-Cell Therapy for the Treatment of Relapsed and Refractory Primary Central Nervous System Diffuse Large B-Cell Lymphoma
Source: Front Oncol. 2019 Dec 4;9:1350. doi: 10.3389/fonc.2019.01350 (PMC6904344; doi:10.3389/fonc.2019.01350)

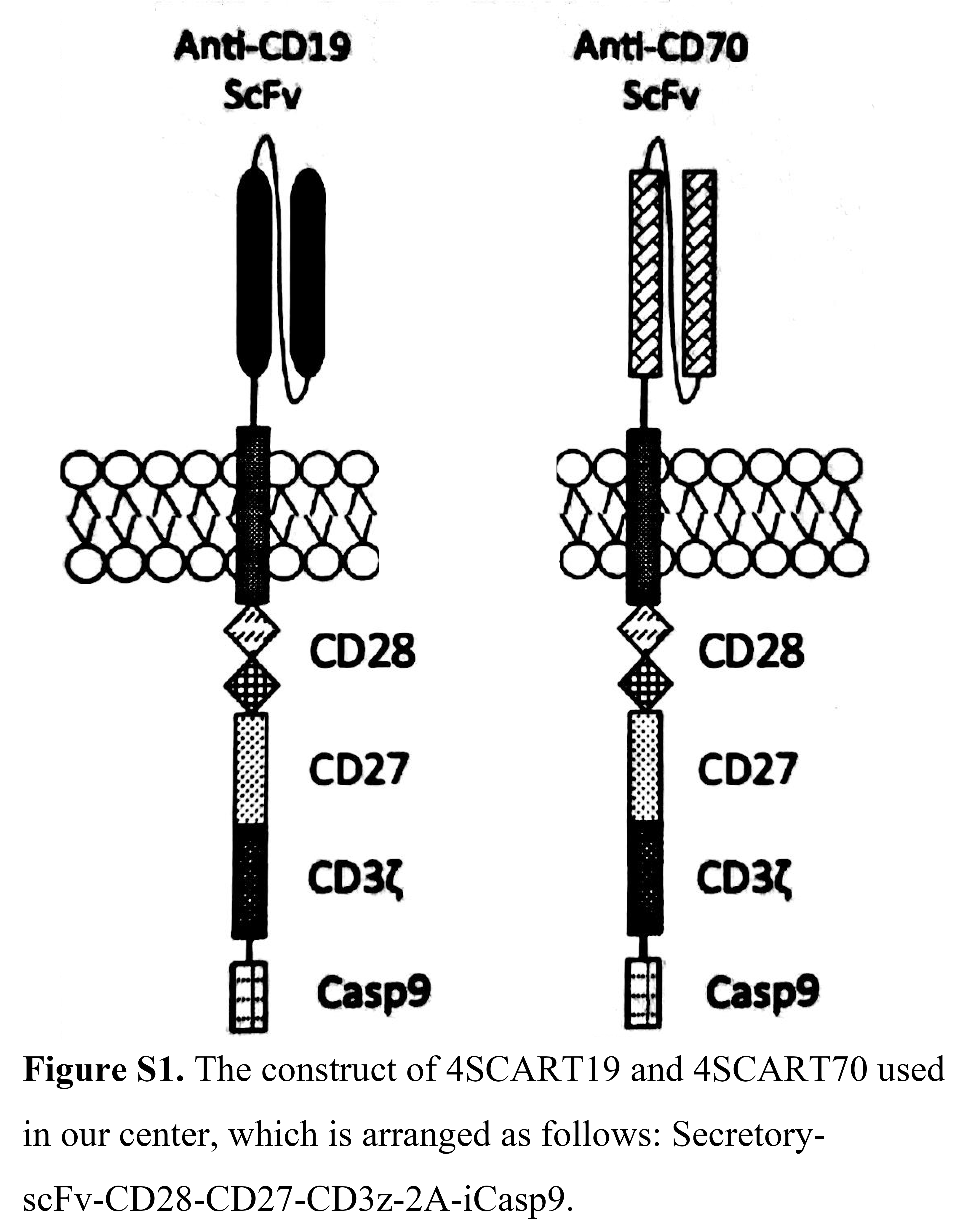

Supplement: Supplementary file 1 [file Image_1.TIF]
